# Quackery in Germany

**Published:** 1898-06

**Authors:** 


### Quackery in Germany.

The *British Medical Journal* says that the Kingdom of Saxony is described by a German medical journal as an Eldorado for quacks. The annual report of the Saxon National Medical College for 1896 states that in that year no fewer than 745 quacks, naked and not ashamed, flourished in that happy land. This number was greater by 42 than that given for the previous year. The total number of legally qualified medical practitioners in Saxony for the corresponding period was 1,761, so that approximately there was one quack to every two regular doctors. In certain districts, however, such as Zittau, Kamenz, Rochlitz, Annaberg, and Glachau, the quacks outnumbered the doctors. Of the 745 quacks, 163 were women. Of the special "lines" of quackery the most numerously represented was "nature healing," which was professed by 220 persons; then came "sympathy," with 106 exponents, homeopathy with 97, massage with 72, "magnetism" with 46, and tape-worm curing with 19. One enterprising gentleman makes a specialty of illumination of the cavities of the body with the electric light.

